# Parameters of Body Composition Predict Clinical Course in Acute Colonic Diverticulitis

**DOI:** 10.1002/jcsm.13864

**Published:** 2025-06-16

**Authors:** Alexey Surov, Mattes Hinnerichs, Iram Shahzadi, Nina P. Haag, Jan Robert Kröger, Berthold Gerdes, Saleem Elhabash, Jan Borggrefe

**Affiliations:** ^1^ Institute of Radiology, Neuroradiology and Nuclear Medicine Johannes‐Wesling‐Klinikum Minden, Ruhr‐University Bochum Bochum Germany; ^2^ Department of Radiology and Nuclear Medicine University Hospital of Magdeburg Magdeburg Germany; ^3^ Department of General Surgery Johannes Wesling University Hospital Minden, Ruhr University Bochum Minden Germany

**Keywords:** acute colonic diverticulitis, myosteatosis, sarcopenia, visceral adiposity

## Abstract

**Background:**

Acute colonic diverticulitis (ACD) is the most common complication of colonic diverticulosis. Body composition, that is, proportion, distribution and quality of muscle and adipose tissues may play a relevant role in ACD. Previously, only few reports with small number of patients analysed the prognostic role of body composition in ACD. Our purpose was to analyse associations between the occurrence of complications and parameters of body composition in patients with ACD in a large sample.

**Methods:**

This retrospective study included 646 patients with ACD. The duration of hospital stay (days) and occurrence of complications were recorded. Parameters of body composition were semiautomatically measured with the freely available ImageJ software. Skeletal muscle area (SMA), skeletal muscle density, visceral adipose tissue (VAT), subcutaneous adipose tissue and intramuscular adipose tissue were estimated. To assess the impact of body composition parameters on ACD complications, Cox regression model (adjusted for sex and age) was used.

**Results:**

Low skeletal muscle area (sarcopenia) was found in 322 patients (49.8%). High VAT was observed in 525 patients (81.3%). Low skeletal muscle density or myosteatosis was identified in 322 patients (49.8%). Length of hospital stay was prolonged in patients with sarcopenia, myosteatosis and/or visceral adiposity. Sarcopenia was an independent predictor for occurrence of complicated ACD, OR = 1.48, 95% CI (1.03–2.13), *p* = 0.033. Myosteatosis predicted occurrence of free perforation, OR = 2.36, 95% CI (1.01–5.43), *p* = 0.033. Furthermore, visceral adiposity tended to be a strong predictor of free perforation, OR = 7.62, 95% CI (1.29–138.00), *p* = 0.05. Finally, sarcopenia predicted occurrence of macro abscesses, OR = 2.41, 95% CI (1.41–4.26), *p* = 0.002.

**Conclusions:**

Patients with sarcopenia, myosteatosis and visceral adiposity have prolonged length of hospital stay. Macro abscesses occur more frequently in patients with sarcopenia. Myosteatosis and high VAT are associated with free perforation.

## 1 Introduction

Acute colonic diverticulitis (ACD) is the most common complication of colonic diverticulosis [1, 2, 3]. According to the literature, up to 25% of patients with colonic diverticulosis develop diverticulitis [1, 2]. Most patients are cured without complications, but 15% undergo severe complications such as abscess formation, bowel perforation and diverticulitis recurrence [1, 2, 3]. Known risk factors for complicated ACD are high C‐reactive protein, high white blood cell count, clinical signs including generalized abdominal pain, constipation and vomiting, steroid usage and comorbidity like diabetes [4].

Contrast‐enhanced computed tomography (CECT) is the diagnostic tool of first choice in patients with suspected diverticulitis [2]. Also, some CT features play a prognostic role in ACD. So far, pericolonic fluid collections and a longer inflamed colon segment are predictive factors on CECT imaging for the progression of uncomplicated into complicated ACD [5].

CT imaging can also provide additionally relevant prognostic data. A detailed body composition, that is, proportion, distribution and quality of muscle and adipose tissues in the body can be characterized by CT [6]. According to the current literature, parameters of body composition play a relevant role in several acute disorders. For example, low skeletal muscle mass or sarcopenia is an important predictor of short‐term mortality in critically ill patients [7]. Low skeletal muscle density or myosteatosis predicts 30‐day mortality in patients with Covid‐19 [8]. Furthermore, sarcopenia and myosteatosis are independent risk factors for mortality among older patients with sepsis in the emergency department [9]. CT‐defined sarcopenia is a risk factor for occurrence of complications in patients with appendicitis [10]. Myosteatosis is associated with poor outcomes in patients with acute pancreatitis [11]. Finally, high visceral adipose tissue (VAT) predicts mortality in patients with cardiogenic shock [12].

In ACD, only few reports with small number of patients analysed the prognostic role of body composition [13, 14, 15].

The purpose of the present study was to analyse associations between the occurrence of complications and parameters of body composition in patients with ACD in a large sample.

## 2 Materials and Methods

This retrospective study was approved by the institutional review board (Ethics Committee of the Faculty of Medicine, Ruhr‐University Bochum, approval code, 2021–827).

### 2.1 Patients

**FIGURE 1:**
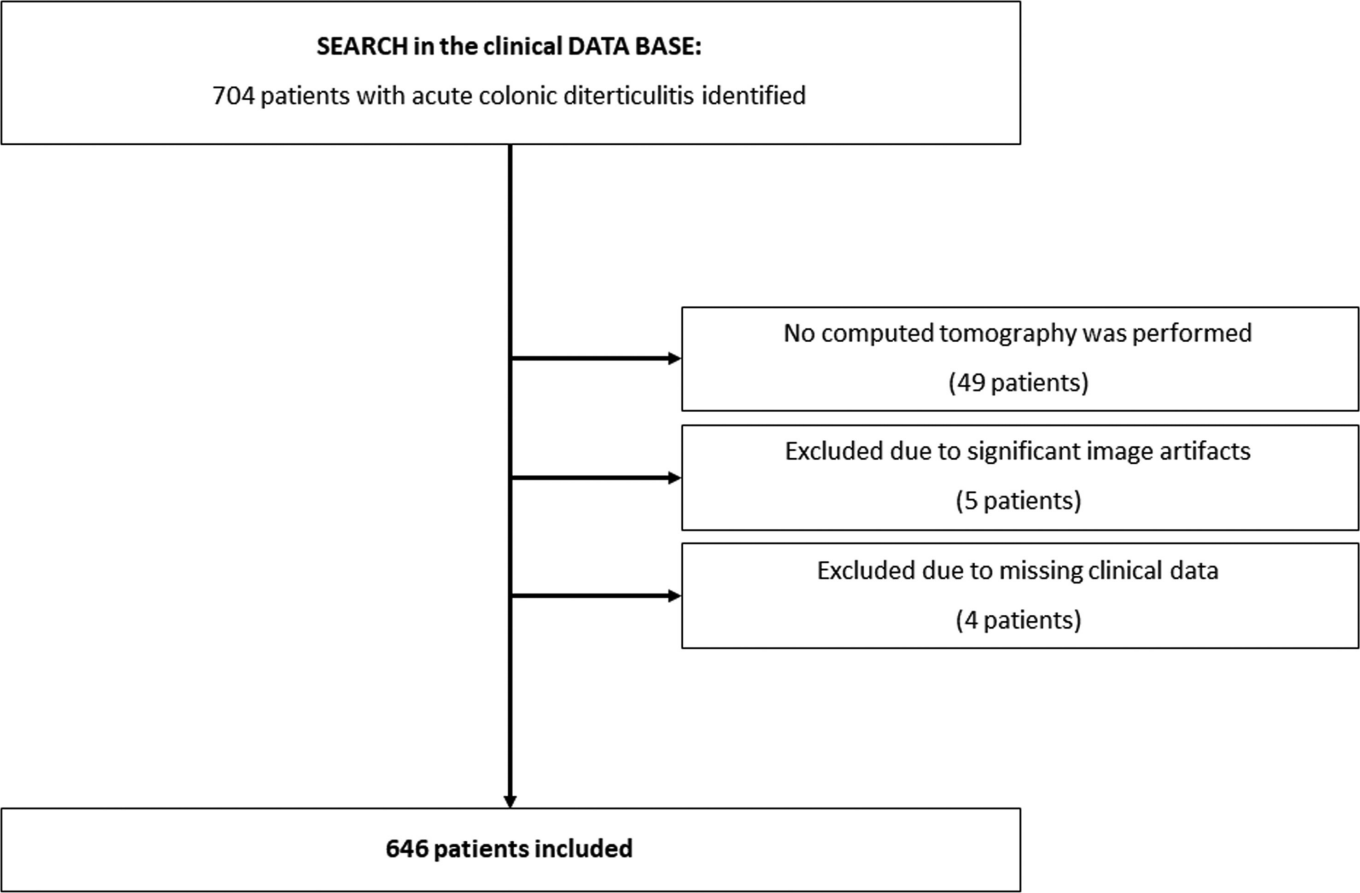
Flow chart diagram of patient acquisition.

All patients with ACD were retrospectively assessed within the time period 2018 to 2024 in our university hospital. A total of 704 patients with ACD was identified in our clinical data base (Figure 1).

Inclusion criteria for the study were the following:

- adult patients of ≥ 18 years of age;
- diagnosis of ACD confirmed by CT;
- available CT images of diagnostic quality.

Exclusion criteria were the following:

- significant artefacts of CT images at the third lumbar level;
- patients with active malignant diseases.

Overall, 58 patients were excluded from the study. The final cohort comprised 646 patients. Medical records were reviewed for clinical and demographic information. The duration of hospital stay (days) and occurrences of complications were recorded.

### 2.2 Image Analysis

**FIGURE 2:**
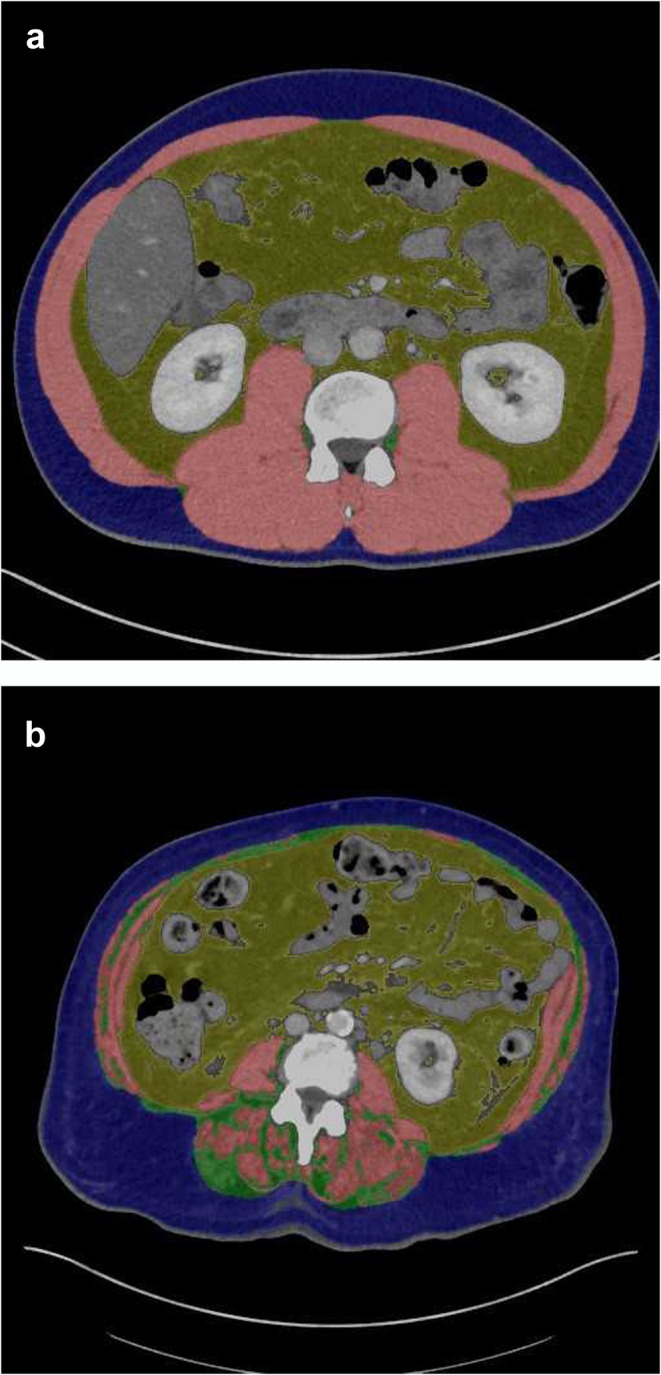
Body composition in two representative patients of the study sample. Staging computer tomographic scans with segmented areas of body composition. Skeletal muscle area (red), intramuscular adipose tissue (yellow), subcutaneous adipose tissue (blue) and intramuscular adipose tissue (green). (a) Patient with normal muscle area and visceral adipose tissue. (b) Patient with low skeletal muscle area (sarcopenia) and high visceral, intramuscular and subcutaneous adipose tissues.

For all patients, CT scans in the portalvenous phase were used (slice thickness of 3 mm). All images were assessed in consensus by two experienced radiologists (M.H. and A.S.), who were blinded to the clinical course of the patients. Parameters of body composition were semiautomatically measured with the freely available ImageJ software (version 1.53, National Institute of Health, USA). One axial slice on the mid of the third lumbar vertebral (L3) was used according to the previous descriptions [16]. Skeletal musculature was estimated with the threshold values of −29 and 150 HU [16]. Skeletal muscle area (SMA) was defined as the cross‐sectional muscle area, including the quadratus lumborum, psoas, rectus abdominus and erector spinae muscles, and the internal transverse and external oblique muscles (Figure 1). For sarcopenia definition, the sex‐specific median SMA values were used as threshold: 156 cm^2^ for male and 103 cm^2^ for female patients. Additionally, radiodensity of the skeletal musculature as a mean attenuation of the included muscles at the L3 level was also estimated. Low muscle radiodensity or myosteatosis was defined as SMD < 41 HU for patients with a body mass index up to 24.9 kg/m^2^ and < 33 HU for patients with a body mass index ≥ 25 kg/m^2^, using the thresholds defined by Martin et al. [17] Fat areas were measured using the HU threshold levels of −190 and −30 HU [18]. VAT and subcutaneous adipose tissue (SAT) were estimated. Furthermore, a ratio of visceral to subcutaneous fat (VSR) was calculated. The proposed threshold value of 100 cm^2^ was utilized as a cut‐off value to determine visceral and/or subcutaneous obesity [18]. High VSR was defined as 1.1. Figure 2a,b displays two representative patients for illustrative purposes.

### 2.3 Statistical Analysis

For statistical analysis, SPSS Version 28 was used (IBM SPSS Statistics for Windows, version 28, Armonk, NY, USA: IBM corporation, 2021). Mean and standard deviation as well as median and interquartile range (IQR) were calculated for continuous variables.

Groups were compared by *χ* ^2^ test for binary outcomes and by *t*‐test for continuous outcomes. To assess the impact of body composition parameters on ACD complications, Cox regression model was used. Odds ratios are presented together with 95% confidence intervals (95% CI). In all instances, *p* values < 0.05 were taken to indicate statistical significance.

## 3 Results

### 3.1 Patients and Parameters of Body Composition

Our sample included 646 patients, 348 female and 298 male with a median age of 61 years (Table 1). In most cases (474, 73.4%) simple diverticulitis was diagnosed. In 172 patients (26.6%), complicated diverticulitis was identified.

**TABLE 1:** Baseline characteristics of the included patients and diagnosed diverticulitis.

Variable	All patients (*n* = 646)
Age in years, median (IQR)	61 (27–98)
Sex, *n* (%)	
Female	348 (53.9)
Male	298 (46.1)
Diverticulitis	
Simple, *n* (%)	474 (73.4)
Complicated, *n* (%)	172 (26.6)
Complicated diverticulitis	
Phlegmon	65 (37.8)
Free perforation	33 (19.2)
Macro abscess	57 (33.1)
Micro abscess	11 (6.4)
Peritonitis	6 (3.5)
Length of hospital stay, days, median (IQR)	5 (0–88)

Low SMA (sarcopenia) was found in 322 patients (49.8%). High intramuscular adipose tissue was identified in 462 patients (72%). High VAT was observed in 525 patients (81.3%). Finally, myosteatosis was identified in 322 patients (49.8%).

### 3.2 Associations Between Body Composition and Complications

Frequency of ACD complications in patients with different parameters of body composition are shown in Table 2. Length of hospital stay was higher in patients with sarcopenia, myosteatosis and/or visceral adiposity (Table 2). Macro abscesses occurred more frequently in patients with sarcopenia in comparison to patients with normal muscle mass (Table 2a). Patients with myosteatosis had a higher proportion of free perforation (*p* = 0.024) (Table 2b). Free perforation was more often diagnosed in patients with visceral adipositas (*p* = 0.041) (Table 2c). Macro abscesses were more frequently found in patients with normal VAT (*p* = 0.042) (Table 2c). There was no difference of ACD subtypes between patients with high and normal IMAT, SAT and VSR (Table 2d–f).

**TABLE 2:** Associations between complications and values of body composition in ACD.

a. Sarcopenia	Sarcopenia	No sarcopenia	*p*s
Simple diverticulitis, *n* (%)	226 (47.7%)	248 (52.3%)	0.082
Complicated diverticulitis, *n* (%)	96 (55.8%)	76 (44.2%)	
LOS, days	8.7 ± 11.31	6.1 ± 6.88	< 0.001
Complicated diverticulitis			
Phlegmon, *n* (%)	28 (29.2%)	35 (46.1%)	0.034
Free perforation, *n* (%)	16 (16.7%)	16 (21.1%)	0.591
Macro abscess, *n* (%)	41 (42.7%)	17 (22.4%)	0.008
Micro abscess, *n* (%)	4 (4.2%)	4 (5.3%)	1.000
Peritonitis, *n* (%)	3 (3.1%)	3 (3.9%)	1.000

b. Myosteatosis	Myosteatosis	No myosteatosis	*p*s
Simple diverticulitis, *n* (%)	232 (48.9%)	242 (51.1%)	0.502
Complicated diverticulitis, *n* (%)	90 (52.3%)	82 (47.7%)	
LOS, days	9.41 ± 11.22	5.47 ± 6.72	< 0.001
Complicated diverticulitis			
Phlegmon, *n* (%)	29 (32.2%)	34 (41.5%)	0.272
Free perforation, *n* (%)	23 (25.6%)	9 (11.0%)	0.024
Macro abscess, *n* (%)	27 (30.0%)	31 (37.8%)	0.358
Micro abscess, *n* (%)	3 (3.3%)	5 (6.1%)	0.619
Peritonitis, *n* (%)	3 (3.3%)	3 (3.7%)	1.000

c. Visceral adipose tissue	High VAT	Normal VAT	*p*s
Simple diverticulitis, *n* (%)	382 (80.6%)	92 (19.4%)	0.535
Complicated diverticulitis, *n* (%)	143 (83.1%)	29 (16.9%)	
LOS, days	7.75 ± 9.622	6.07 ± 8.52	0.034
Complicated diverticulitis			
Phlegmon, *n* (%)	55 (38.5%)	8 (27.6%)	0.370
Free perforation, *n* (%)	31 (21.7%)	1 (3.4%)	0.041
Macro abscess, *n* (%)	43 (30.1%)	15 (51.7%)	0.042
Micro abscess, *n* (%)	6 (4.2%)	2 (6.9%)	0.884
Peritonitis, *n* (%)	3 (2.1%)	3 (10.3%)	0.099

d. Intramuscular adipose tissue (IMAT)	High IMAT	Normal IMAT	*p*s
Simple diverticulitis, *n* (%)	338 (71.30%)	136 (28.69%)	0.922
Complicated diverticulitis, *n* (%)	124 (72.09%)	48 (27.91%)	
LOS, days	7.65 ± 9.27	6.88 ± 9.86	0.148
Complicated diverticulitis			
Phlegmon, *n* (%)	40 (32.3%)	23 (47.9%)	0.083
Free perforation, *n* (%)	24 (19.4%)	7 (14.6%)	0.611
Macro abscess, *n* (%)	44 (35.5%)	12 (25.0%)	0.256
Micro abscess, *n* (%)	0 (0.0%)	0 (0.0%)	1.000
Peritonitis, *n* (%)	3 (2.4%)	3 (6.2%)	0.444

e. Subcutaneous adipose tissue (SAT)	High SAT	Normal SAT	*p*s
Simple diverticulitis, *n* (%)	447 (94.3%)	27 (5.7%)	0.536
Complicated diverticulitis, *n* (%)	165 (95.9%)	7 (4.1%)	
LOS, median days	5[0–88]	5.5[0–80]	0.948
Complicated diverticulitis			
Phlegmon, *n* (%)	60 (36.4%)	3 (42.9%)	1.000
Free perforation, *n* (%)	32 (19.4%)	0 (0.0%)	0.426
Macro abscess, *n* (%)	55 (33.3%)	3 (42.9%)	0.909
Micro abscess, *n* (%)	8 (4.8%)	0 (0.0%)	1.000
Peritonitis, *n* (%)	5 (3.0%)	1 (14.3%)	0.591

f. Ratio of visceral to subcutaneous fat (VSR)	High VSR	Normal VSR	*p*s
Simple diverticulitis, *n* (%)	143 (30.2%)	331 (69.8%)	0.186
Complicated diverticulitis, *n* (%)	62 (36.0%)	110 (64.0%)	
LOS, median days	5 [0–88]	5[0–80]	0.074
Complicated diverticulitis			
Phlegmon, *n* (%)	26 (41.9%)	37 (33.6%)	0.358
Free perforation, *n* (%)	10 (16.1%)	22 (20.0%)	0.673
Macro abscess, *n* (%)	18 (29.0%)	40 (36.4%)	0.419
Micro abscess, *n* (%)	2 (3.2%)	6 (5.5%)	0.772
Peritonitis, *n* (%)	2 (3.2%)	4 (3.6%)	1.000

Regression analyses (Table 3a–c) identified that sarcopenia was an independent predictor for complicated ACD, OR = 1.48, 95% CI (1.03–2.13), *p* = 0.033. Myosteatosis predicted occurrence of free perforation, OR = 2.36, 95% CI (1.01–5.43), *p* = 0.033. Furthermore, high VAT tended to be a strong predictor of free perforation, OR = 7.62, 95% CI (1.29–138.00), *p* = 0.05. Finally, sarcopenia predicted occurrence of macro abscesses, OR = 2.41, 95% CI (1.41–4.26), *p* = 0.002 (Table 3c).

**TABLE 3:** Prediction of complications in patients with ACD.

	Univariable			Multivariable		
Variables	OR	95% CI	*p*	OR	95% CI	*p*
a. Prediction of complicated disease						
Age (years), > 60 (vs. < 59)	0.733	0.515–1.04	0.082	0.692	0.480–0.993	0.046
Sex (male vs. female)	0.736	0.518–1.04	0.085	0.768	0.539–1.09	0.144
Sarcopenia (vs. vs. normal muscle area)	1.39	0.977–1.97	0.068	1.48	1.03–2.13	0.033
High VAT (vs. normal VAT)	1.19	0.758–1.91	0.463			
Myosteatosis (vs. normal muscle density)	1.14	0.808–1.62	0.448			
High IMAT (vs. normal IMAT)	1.04	0.708–1.54	0.845			
High SAT (vs. normal SAT)	1.42	0.641–3.61	0.415			
High VSR (vs. normal VSR)	1.30	0.900–1.88	0.157			
b. Prediction of free perforation						
Age (years), > 60 (vs. < 59)	0.601	0.029–1.22	0.163	0.437	0.203–0.910	0.029
Sex (male vs. female)	1.34	0.660–2.80	0.427	1.72	0.835–3.66	0.146
Sarcopenia (vs. normal muscle area)	1.07	0.530–2.18	0.844			
High VAT (vs. normal VAT)	7.79	1.65–139.00	0.044	7.61	1.29–138.00	0.05
Myosteatosis (vs. normal muscle density)	2.42	1.16–5.39	0.023	2.36	1.01–5.43	0.033
High IMAT (vs. normal IMAT)	1.51	6.77–3.83	0.345			
High VSR (vs. normal VSR)	0.932	0.417–1.94	0.856			
c. Prediction of macro abscesses						
Age (years), > 60 (vs. < 59)	1.19	0.718–1.99	0.500	1.00	0.593–1.70	0.998
Sex (male vs. female)	1.06	0.640–1.78	0.814	1.06	0.635–1.79	0.822
Sarcopenia (vs. vs. normal muscle area)	2.40	1.42–4.21	0.001	2.41	1.41–4.26	0.002
High VAT (vs. normal VAT)	0.644	0.363–1.19	0.144			
Myosteatosis (vs. normal muscle density)	0.852	0.511–1.41	0.537			
High IMAT (vs. normal IMAT)	1.57	0.873–3.02	0.149			
High SAT (vs. normal SAT)	1.21	0.416–5.31	0.761			
High VSR (vs. normal VSR)	0.980	0.558–1.67	0.942			

## 4 Discussion

Some previous studies also show relevant relationships between body composition and clinical course in ACD. However, the reported results are conflicting. Furthermore, most of them analysed only the prognostic role of adipose tissue in ACD. For example, according to Dahlbäck et al., there are no associations of the skeletal muscle quantity and/or quality with complications in patients with ACD [19]. However, myosteatosis is associated with prolonged hospital stay [19]. In the study of Schaffler‐Schaden, skeletal muscle mass does not predict occurrence of complications in ACD [20]. Furthermore, Jeong et al. suggest that visceral obesity is significantly associated with complications of diverticulitis [21]. In the study of Ng et al., however, none of adipose tissue values differs between patients with complicated and uncomplicated ACD [15].

Our results show strong associations between body composition and occurrence of complications in ACD. Also our data suggest that these associations are complex and different in several parameters of body composition. So far, myosteatosis is a strong and independent predictor of free perforation. Also patients with high VAT show more frequently free perforation. Finally, sarcopenia is strongly associated with occurrence of macro abscesses. No relevant associations were found between complications and VSR and/or SAT.

The identified findings may guide patient management and/or therapeutic interventions.

Furthermore parameters of body composition, namely LSMM, myosteatosis and high VAT are associated with prolonged length of hospital stay.

The identified relationships between body composition and complications in patients with ACD are multicausal. First, low muscle mass is associated with malnutrition. Patients with malnutrition have a worse general performance including anaemia. Second, the identified effects of the reduced muscle mass and quality may be explained by the endocrine function of the skeletal musculature. Skeletal muscles produce and release several cytokines, so called myokines, which influence almost all system and organs. More importantly, some myokines have anti‐inflammatory effects [22, 23]. For instance, the myokine irisin decreases significantly several inflammatory markers and the histological changes of the intestinal mucosa in experimental colitis [22]. Low muscle mass and quality may influence production of myokines.

Visceral fat is also a metabolic active tissue that produces numerous pro‐inflammatory cytokines [24]. So far, VAT expresses inflammatory peptides, including interleukin 1β, interleukin 6 and tumour necrosis factor α, which trigger inflammation [24]. Furthermore, VAT synthetizes and realizes adipokines with local proinflammatory effects. For example, VAT produces resistin, an adipokine that stimulates immune cells [24]. Another adipokine chemerin promotes the chemotaxis of dendritic cells and macrophages and acts as a chemoattractant for cells of innate immunity [25]. Patients with high proportion of VAT may have a higher serum level of proinflammatory substances.

Our study has limitations due to its retrospective nature. However, our results are based on the largest cohort to date. Furthermore, we used CT defined parameters of body composition only.

In conclusion, in ACD, patients with sarcopenia, myosteatosis and visceral adipositas have prolonged length of hospital stay. Macro abscesses occur more frequently in patients with sarcopenia. Myosteatosis and high VAT are associated with free perforation.

## Conflicts of Interest

The authors declare no conflicts of interest.
